# Health Literacy and Interventions on Antibiotics Use and AMR in Younger Generations in High-Income Countries—A Systematic Review

**DOI:** 10.3390/antibiotics14090940

**Published:** 2025-09-17

**Authors:** Katja Molan, Anamarija Zore, Nevenka Kregar Velikonja

**Affiliations:** 1 Faculty of Health Sciences, University of Novo mesto, Na Loko 2, 8000 Novo mesto, Slovenia; katja.molan@uni-nm.si; 2 Faculty of Health Sciences, University of Ljubljana, Zdravstvena pot 5, 1000 Ljubljana, Slovenia; anamarija.zore@zf.uni-lj.si

**Keywords:** antibiotic use, AMR, health literacy, educational interventions, young people, students, high-income countries (HICs)

## Abstract

Antimicrobial resistance (AMR) is a growing threat to global health, accelerated by the widespread inappropriate use of antibiotics. Although educational initiatives have been launched worldwide, there is little evidence on how younger generations in high-income countries (HICs) understand and address AMR. Addressing the AMR crisis requires proactive education of younger generations, including children, adolescents, and young adults, who will shape future healthcare practices. This review analyzes existing research on AMR literacy among these age groups in HICs, as knowledge gaps and risky behaviors persist even in HICs, despite their strong education and health infrastructures. The purpose of this review is to examine the knowledge, attitudes, and behaviors related to antibiotic use and antibiotic resistance in younger generations while identifying effective educational interventions. Methods: We performed a comprehensive literature search in PubMed until June 2025, followed by AI-assisted screening (Claude 4.0 Sonnet) and a manual review. The search strategy combined terms from the areas of health literacy, antibiotics, antibiotic resistance/AMR, and young populations. Studies in HICs that examined the younger generation’s knowledge about antibiotics and AMR, analyzed their attitudes or behavior toward them, or evaluated relevant educational interventions were included. Data were synthesized thematically across all included studies. Results: Nineteen studies from 11 HICs were included, including thirteen cross-sectional surveys and six educational intervention studies. The results showed that misconceptions about how antibiotics work are still very common. Several of those asked (22–80%) incorrectly stated that resistance develops in the human body and not in bacteria. Many (26–77%) mistakenly agreed with the statement that antibiotics treat viral infections. Concerning behaviors included high rates of self-medication, non-adherence to treatment, and unsafe storage practices. Several authors propose an amendment of curricula. Educational interventions, particularly gamification and peer education approaches, showed improvements in knowledge and sustained learning outcomes. Conclusions: Knowledge of AMR among young people in HICs is still inadequate, despite educational advantages. Most existing studies focus on college students, while children and adolescents, crucial groups for early prevention, are underrepresented. Targeted, age-appropriate education employing interactive methods represents an evidence-based strategy to improve antibiotic use behavior and support global AMR control efforts.

## 1. Introduction

Antibiotic resistance of bacteria/antibiotic microbial resistance (AMR) is one of the most serious global health problems and threatens the sustainability of the global response to infectious diseases [1,2,3]. AMR occurs when microorganisms develop resistance to antibiotic treatment and no longer respond to antimicrobial drugs [1,2,3,4,5,6]. The World Health Organization (WHO) and the European Center for Disease Prevention and Control (ECDC) report an increase in antimicrobial resistance [7]. The spread of antimicrobial resistance mechanisms is also leading to a generation of multidrug-resistant (MDR) organisms, characterized by resistance to multiple classes of drugs. Infections caused by MDR microorganisms are associated with increased mortality, longer hospital stays, and elevated healthcare costs [8]. This growing global public health crisis is driven in large part by the misuse and overuse of antibiotics in both healthcare and community settings. The WHO has published a report on the monitoring of the global strategy to combat antimicrobial resistance [9], which emphasizes the importance of raising public awareness of the risks associated with MDR and training health, veterinary, and agricultural staff. Many countries have adopted the “One Health” action plan for the control of AMR [10], which sets out the priority areas for action, including educating professionals and the general public and thus improving health literacy.

Health literacy (HL) is defined as “the extent to which individuals are able to obtain, communicate, process and understand basic health information in order to make appropriate health decisions” [11]. This understanding of health literacy is based on a shared responsibility between the users and the actors of the health system, in which adults are the main actors [12]. Today, the meaning of the term seems to be more complex and heterogeneous than ever [11,13,14]. With respect to children and adolescents, it may not be sufficient to capture health literacy as a two-sided concept [15]. Adolescent health literacy is critical for navigating the complex healthcare landscape and making informed decisions that influence long-term health outcomes [16]. Young people aged 13 to 26 are in a critical developmental period during which they form attitudes and behaviors that often persist into adulthood. Health literacy interventions must be designed with the needs of the target groups in mind. However, children and adolescents are often addressed as part of the general population [12]. Addressing AMR demands long-term, sustained action; therefore, the younger generation is an important target group for AMR education and awareness, as it will play a key role in shaping the future of healthcare [17]. Assessing and strengthening health literacy on AMR among young people is crucial to prevent the escalation of this public health crisis. A thorough examination of why younger generations, especially those that are well-educated, report high rates of antibiotic misuse is warranted [18,19,20,21,22]. Assessing the health literacy of younger generations in relation to AMR is about assessing their understanding of key concepts, their ability to make informed decisions about antibiotic use, and their general awareness of the impact of drug resistance [12,17].

Although AMR-related problems should be addressed as a global issue, there are several contextual differences between HICs and low- and middle-income countries (LMICs) in terms of antibiotic use and policies to prevent AMR from spreading [23,24,25]. Although it is often assumed that antibiotic problems are more acute in low-income countries, AMR is also a major and growing problem in HICs, often due to the overuse of antibiotics in human and veterinary medicine, as well as in agriculture [1,2,4]. HICs possess the financial and institutional capacity to invest in research, innovation, and policy implementation related to AMR. While higher levels of health literacy and AMR-related preventive behaviors might be expected in HICs due to technological advancement and well-resourced healthcare and educational systems, studies show that knowledge among young people in HICs remains suboptimal [26,27,28,29,30]. As highlighted by Dambrino and Green [19], perceptions and practices vary among young people in HICs. Given that this population represents future healthcare users, decision-makers, and health professionals, understanding existing approaches to education and raising awareness is crucial.

The primary objective of this review is to systematically synthesize current evidence on AMR literacy, including knowledge, attitudes, and behaviors, among younger generations in HICs. For the purposes of this review, we use the umbrella term “younger generations” to refer to children (6–12 years), adolescents (13–18 years), and young adults (19–24 years), in line with common age categorizations in health literacy research [14]. When discussing individual studies, we specify the exact population examined. A secondary objective is to identify effective educational interventions designed to improve AMR literacy in this population. By summarizing current evidence, this article attempts to highlight effective approaches to improve awareness and promote responsible antibiotic use in the younger generations, thus contributing to global efforts to mitigate the AMR threat.

## 2. Results

**Figure 1:**
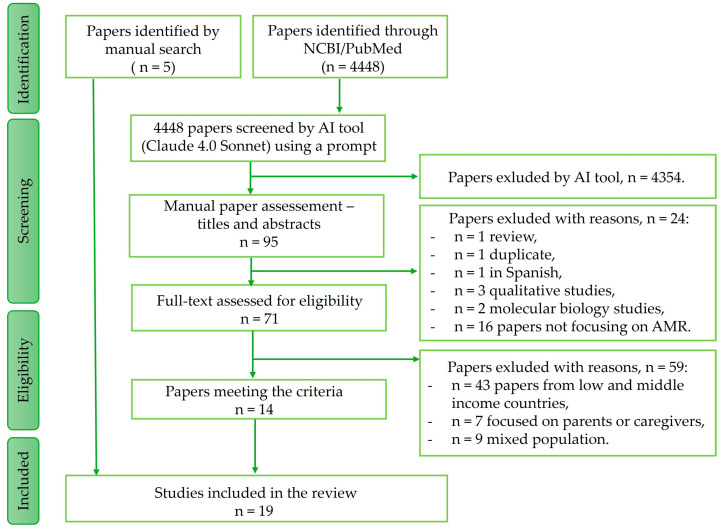
Prisma flow chart.

For this review, 19 studies investigating antibiotic- and AMR-related health literacy among young generations in HICs were identified and included in the analysis. The method of article selection is explained in the Methods section (Figure 1). Of the 19 studies, 13 (68.5%) were cross-sectional surveys (Table 1) and 6 (31.5%) were educational intervention studies with pre–post evaluation (Table 2).

Table 1 summarizes the 13 observational studies that examined the baseline knowledge, attitudes, and behaviors related to antibiotic use and AMR among younger populations in HICs. This comprehensive selection, which included papers from 10 countries, provides a robust foundation for understanding antibiotic health literacy gaps among young generations in HIC. The table presents each study’s title, authors, country, study population, and main findings regarding AMR literacy (including general awareness, misconceptions, non-adherence, and self-medication), along with the authors’ proposed educational interventions.

The synthesis of the findings from the selected articles is focused on three main themes to highlight/emphasize: general AMR awareness and fundamental misconceptions and gaps in knowledge about antibiotic mechanisms and resistance; self-medication and non-adherence behaviors; and suggested educational interventions and strategic approaches.

### 2.1. General AMR Awareness and Misconceptions and Gaps in Knowledge About Antibiotic Mechanisms and Resistance

There are persistent and widespread misconceptions among the young population about the basic mechanisms of antibiotics and the concepts of antimicrobial resistance. The most important misconception is confusion about where resistance originates from. Surveys in Canada [36], the UK [32], and Italy [31] show that a large proportion of students (22–78%) confuse bacterial resistance with human or animal resistance. Similar misconceptions were also found among senior secondary school students in the Middle East [29].

The misconception that antibiotics are effective against viral infections is another common knowledge gap. Surveys in the Middle East [21,27,28,29] demonstrate a high level (30–70%) of this misconception among senior secondary school students, as well as non-medical and medical students. Relatively high levels of this misconception were also reported among medical students from Italy (20%) [35]. Hagiya et al. [33] showed that misconceptions about the effectiveness of antibiotics against viral diseases decrease significantly as medical education progresses. Significant differences between first-year medical students and advanced students, with advanced students showing significantly better knowledge in all areas, were also reported in a study from Germany [34].

Among those pursuing a career in healthcare, there are worrying gaps. In a study from Saudi Arabia, it was reported that only 71.8% of medical students were aware of the term “antibiotic resistance”, but many did not understand the concept correctly as their answers reflected also various misconceptions: 57.7% thought paracetamol was an antibiotic and 58.8% believed antibiotics could replace anti-inflammatory drugs [27]. Further, in a study among Japanese medical students, better general knowledge was revealed, as 92.6% knew that antibiotics inhibit bacterial growth, but there were misconceptions regarding the use of antibiotics as an option for the treatment of the common cold and other viral diseases (up to 46%) [33]. Wiese-Posselt et al. [34] reported that only 5% of German medical students felt they had gained sufficient knowledge of antibiotic therapy and antibiotic resistance during their studies, and only 54% recognized the relevance of antibiotic resistance to clinical practice.

### 2.2. Self-Medication and Non-Adherence Behaviors

Risky self-medication practices are prevalent in diverse student populations and represent a direct pathway to resistance development. A total of 28.3% of Canadian students had taken antibiotics without a prescription, obtaining them from home medicine cabinets (16.7%), pharmacies (50%), family/friends (26.7%), or other sources (6.7%) [36]. Among US students, 23% would take antibiotics that were not prescribed to them, while 13% would share their antibiotics with others [30]. Among German medical students, only 10% had taken antibiotics without a prescription by obtaining them themselves, buying them abroad, or using leftover medication [34], and 16% of surveyed Italian students had bought antibiotics without a prescription [35]. Benameur et al. [22] found that while 79.9% of Saudi medical students knew self-medication was unsafe, 50.8% still practiced it.

Non-completion of treatment is another important behavioral risk factor. Across countries, between one-third and one-half of students reported stopping antibiotics early, often when symptoms improved or due to side effects [28,29,30,33]. Adherence was higher among European medical students, with studies from Italy and Germany reporting lower discontinuation rates (8–15%) [34,35].

Studies identified concerning patterns of antibiotic storage and sharing. Abu-Mostafa et al. [18] found that 47.8% of students stored antibiotics at home for later use. Benameur et al. [22] reported that 40.8% of medical students and 47.6% of non-medical students expressed a negative attitude towards storing leftover antibiotics, suggesting that a significant proportion considered this practice acceptable. A total of 7.7% of Italian medical students normally used leftover antibiotics without consulting doctors and 16% bought antibiotics without prescription [35].

### 2.3. Suggested Educational Interventions and Strategic Approaches

Authors of cross-sectional studies, presented in Table 1, consistently recommend tailored intervention strategies that differ between medical students and the general young population, reflecting their different educational needs and professional responsibilities. Interventions for general young populations include broader public health approaches and integration of AMR-related contents into the curriculum. Changes in the curriculum to address antibiotic misconceptions are proposed on the secondary school level, as well as on the level of undergraduate study programs, especially in the field of medicine and health sciences [21,22,26,27,33,34,35]. Educating students about the science of antibiotic resistance is crucial to empower them with self-efficacy for antimicrobial stewardship behaviors [30]. Authors often emphasize the active role of students in the education process. Akbar et al. [27] recommended seminars and training to improve awareness. Also, early involvement of medical students in the antimicrobial stewardship program, not only as active learners but also as active medical team players, is recommended [22]. Addressing non-medical students by regular community campaigns to educate students about the indications, efficacy, and side effects of antibiotics has also been suggested. Abu-Mostafa et al. [28] suggested conducting community outreach campaigns every university semester and recommended that health professionals should participate in education. Also, Tran et al. [30] emphasized that campus health services can play a critical role in educating all students about the safe use of antibiotics. Prigitano et al. [31] argued for the promotion of initiatives that emphasize how to prevent antibiotic resistance through appropriate use, completing prescribed treatments and prevention of misuse.

**Table 2:** Studies with intervention aiming to improve AMR literacy.

Title (Author, Year)	Country	Population (N and Major Characteristics)	Study Type	Intervention	Main Findings Regarding Antibiotic-/AMR-Related Knowledge/Behavior
Students’ perceptions of a blended learning pharmacy seminar course in a Caribbean school of pharmacy (Extavour and Allison 2017) [37]	Trinidad and Tobago	37 pharmacy students at a Caribbean School of Pharmacy, 21–49 years	Post intervention evaluation study	Blended learning effective for critical thinking	Measurement of perceptions regarding blended learning: theme of antibiotic resistance ranked as 2nd most helpful topic.
Enhancing Medical Students’ Confidence and Knowledge in Antibiotic Prescription and Administration through Virtual Education: A Quasi-Experimental Study (Malli et al., 2023) [38]	Saudi Arabia	33 medical students, mean age 22.6 ± 2.17	Quasi-experimental study	WHO online antibiotic stewardship course	The group’s baseline confidence and background knowledge were lower than after introducing the virtual course.
‘The Mould that Changed the World’: Quantitative and qualitative evaluation of children’s knowledge and motivation for behavioural change following participation in an antimicrobial resistance musical (Hall et al., 2020) [39]	UK, Scotland	182 children, aged 9 to 11 years	Mixed-methods pre and post intervention evaluation	The primary school musical *The Mould that Changed the World*	Musical theater improved short and long-term knowledge. It demonstrated attitude and motivation to change behavior in children at an influential age for health beliefs.
Can Gaming Increase Antibiotic Awareness in Children? A Mixed-Methods Approach (Hale et al., 2017) [40]	UK, England	153 pupils aged 9–11 years	Mixed-methods pre- and post-intervention evaluation	Gamification using e-Bug games for a total of 15 min	Body Busters game most effectively increased antibiotic knowledge in children and had the greatest flow and enjoyment.
A mixed-method evaluation of peer-education workshops for school-aged children to teach about antibiotics, microbes and hygiene (Young et al., 2017) [41]	UK, England	476 secondary (12–13 years old) and 589 primary (9–11 years old) school students	Mixed-methods pre- and post-intervention evaluation, retention evaluation	Peer education interventions (workshops)	Significant improvement in both primary and secondary students; peer educators retained more knowledge.
Aston University’s Antimicrobial Resistance (AMR) Roadshow: raising awareness and embedding knowledge of AMR in key stage 4 learners (Ahmed et al., 2020) [42]	UK, England	159 students; the majority of students attending the live performance were within ages 14–16	Quasi-experimental with pre- and post-intervention evaluation, retention evaluation	An interactive session under AMR Roadshow event	The roadshow significantly improved knowledge and understanding of AMR, which was retained for a minimum of twelve weeks.

The six interventional studies that focused on various educational approaches, including gamification, blended learning, musical intervention, the WHO online antibiotic stewardship course, a peer education intervention (workshops), and an interactive session, are presented in Table 2. The analysis included papers from three HICs. The reported details include the study title, authors, country, study population, design, type of intervention, and outcomes in terms of immediate knowledge gains and sustained learning effects.

Across the studies, different interventions and educational approaches for teaching antibiotic resistance and antibiotic stewardship to young populations were proposed and evaluated:

1. Peer education: Young et al. [41] conducted a study with a mixed methodological approach with peer education workshops in four schools in England, where secondary school students (12–13 years) taught primary school students (9–11 years) about antibiotics, microbes, and hygiene. Both primary and secondary school students showed a significant increase in knowledge before and after the intervention, with peer educators retaining more knowledge over time.
2. Gamification: Hale et al. [40] conducted a study with a mixed methodological approach and evaluated three e-Bug educational games (Doctor Doctor, Microbe Mania, and Body Busters) with children aged 9–11 years in the UK. The Body Busters game most effectively increased antibiotic knowledge in children and had the greatest flow and enjoyment.
3. Art pedagogics: Hall et al. [39] conducted a study with a mixed methodological approach with the primary school musical *The Mould that Changed the World*. Musical theater improved short- and long-term knowledge. It improved the attitudes of children and motivation for behavior change in children at an influential age regarding health beliefs.
4. Online events/courses: Malli et al. [38] performed a quasi-experimental study and assessed the WHO online antibiotic stewardship course with medical students in Saudi Arabia. The group’s baseline confidence and background knowledge were lower than those after introducing the virtual course. Extavour and Allison [37] evaluated blended learning approaches in pharmacy education at a Caribbean college combining face-to-face and online activities. An interactive AMR roadshow with four workshops for students aged 14–16 was evaluated, where knowledge and subsequent retention were measured pre- and post-event through a standardized questionnaire. It was reported that knowledge and attitudes improved immediately after the intervention and that knowledge was maintained for at least twelve weeks [42].

Interventions were applied to diverse populations, including primary and secondary school children, pharmacy and medical students, and the general student population. A diverse range of innovative educational approaches consistently demonstrated positive effects on AMR-related knowledge, attitudes, and behaviors.

## 3. Discussion

The present review demonstrates that health literacy regarding antimicrobial resistance (AMR) among young population groups in high-income countries (HICs) remains suboptimal. Despite available educational resources, evidence consistently indicates insufficient knowledge and inappropriate behaviors related to antibiotic use across younger generations, irrespective of geographic or educational context. Proposed interventions to enhance AMR-related knowledge and practices include the integration of revised curricular content, alongside the implementation of evidence-based strategies such as gamification, peer-led education, and the use of digital tools with measurable improvements.

### 3.1. Knowledge Gaps and Inappropriate Antibiotic Use Behaviors

Analysis of the cross-sectional studies in HICs confirms two consistent patterns: widespread misconceptions about the basic mechanisms of antibiotics and resistance and risky behaviors associated with antibiotic use. In the included countries and educational institutions, the misconception that antibiotics can treat viral infections is widespread [21,26,28,29,30,33,34,35,36], as is the misconception that resistance develops in the human/animal body and not in bacteria [21,27,28,29,32,33,36]. These misconceptions have been found not only among secondary school students but also among medical and pharmacy students, suggesting that existing curricula do not adequately address such fundamental misunderstandings. This challenges the assumption that an accessible and well-resourced educational and health care system, enabling educational and health prevention campaigns, leads to appropriate antibiotic use behavior. In particular, the prevalent misconception that antibiotic resistance develops within the human body rather than in bacteria underscores a critical gap in the teaching of core scientific concepts.

A comprehensive systematic review from 2025, analyzing global data, reported that self-medication with antibiotics affects 43% of young people worldwide, with students representing the largest risk group [43]. Self-medication among both medical and non-medical students was reported in the selected studies [22,28,30,34,35,36]. This discrepancy is particularly pronounced among medical students, who, although they have above-average theoretical knowledge, still practice self-medication and non-adherence. This phenomenon suggests that training programs are prioritizing the acquisition of clinical knowledge over the development of personal skills. The fact that medical students recognize the professional ethic of appropriate prescribing while practicing inappropriate personal use of medications indicates a compartmentalization of knowledge that needs to be explicitly addressed in education. Among the studies listed in Table 1, Middle Eastern countries report the highest self-medication rates, up to 50%, versus up to 16% in reported in two studies from European countries [34,35]. In Saudi Arabia, easy antibiotic access and family influence drive this behavior [44], while in Europe, stricter regulations, accessible care, and higher trust in medical professionals reduce it [45]. Marked variability was observed across countries and study groups. Misconceptions about antibiotics treating viral infections ranged from 1% among UK medical students [32] to nearly 70% among Saudi health students [27], while misunderstandings of resistance mechanisms ranged from 22% in Italy [35] to 63.2% in Canada [36]. These differences point to the influence of cultural, educational, and systemic factors, highlighting the need for context-specific interventions. Our results suggest that AMR-related misconceptions are reinforced by informal social networks, which often outweigh the influence of formal education. Students reported receiving antibiotics from family or friends, indicating that family plays a central role in the transmission of health-related behaviors. It is well established that keeping antibiotics at home greatly increases the likelihood of self-medication and that parental practices strongly influence young people’s use and awareness [46,47]. Effective AMR interventions therefore need to involve families and peer networks, not just selected school populations.

Although medical students generally outperform their non-medical peers in terms of antibiotic- and AMR-related knowledge and health literacy [20,21,22], persistent misconceptions remain, and many do not feel adequately trained. A multi-center cross-sectional study focusing on responsible antibiotic prescription including 29 European countries showed that over a third of the medical students wanted more education on antimicrobial use with northern countries showing a higher amount of confidence [48]. This may reflect deeper problems with traditional educational approaches that treat AMR as a purely biological or medical problem rather than a complex behavioral and social challenge. The persistence of misconceptions across geographic and institutional contexts in HICs points to a systemic rather than isolated failure in education and highlights the need for a fundamental paradigm shift in the way AMR education is conceptualized and delivered. The AMR theme requires not only an earlier introduction of core concepts in school but also their systematic reinforcement throughout higher education, particularly in the health sciences [49]. Without greater integration into curriculum, misconceptions and risky behaviors are likely to persist in practice, weakening efforts to curb antimicrobial resistance.

The persistent gap between knowledge and behavior observed in this review aligns with behavior change theories, which show that knowledge alone rarely drives lasting change. Theories like the Theory of Planned Behavior [50] and Social Cognitive Theory [51] emphasize addressing motivation, self-efficacy, and social norms alongside cognitive understanding. In AMR stewardship, this means educational programs should reinforce key concepts repeatedly, provide ongoing support to prevent relapse, and target personal attitudes toward antibiotic use rather than relying solely on information delivery. Interactive approaches such as gamification and peer education succeed because they engage motivation, offer repeated exposure through diverse modalities and create social accountability, providing a more comprehensive route to sustainable antibiotic stewardship among younger generations.

### 3.2. Targeted Interventions Aimed at Improving Antibiotic- and AMR-Related Knowledge, Attitudes, and Behaviors

Targeted educational interventions can significantly improve antibiotic knowledge among school and university students when the educational content is specifically designed for these populations [39,40,52,53]. Young et al. [41] showed that peer-to-peer teaching not only raised knowledge among younger students but also enhanced retention among the student tutors, with effects persisting at six weeks, though they were attenuated compared with immediate post-intervention levels. Peer education promotes deeper learning through active engagement and consistently enhances tutors’ understanding and skills, through processes like preparing, explaining, and giving feedback [54]. Extavour and Allison [37] evaluated blended learning approaches in pharmacy education at a Caribbean college, where students participated in seminars that combined face-to-face and online activities, with antibiotic resistance being the second most helpful topic for students. All learning resources were rated as generally helpful. At a US university, an interactive origami fortune teller tool, with various case scenarios to demonstrate key antibiotic principles, used by peer educators, significantly raised student assessment scores, showing positive trends in increasing student AMR stewardship knowledge [55]. This aligns with the educational theory suggesting that interactive and experiential learning promotes higher-order cognitive processing and longer-term retention compared to didactic instruction [56,57].

Creative pedagogy is a broad educational approach that emphasizes innovation, imagination, and non-traditional methods to enhance learning. It encourages students to think creatively and engage with content in active, meaningful ways. Hall et al. [39] evaluated the efficiency of arts integration (through a musical), which is an educational approach in which artistic processes are intentionally woven into the teaching and learning of non-art subjects (such as science) to deepen understanding and improve learning outcomes. The study revealed that attitude and motivation to change behavior in children at an influential age for health beliefs. Another example of a creative approach is gamification. Studies show that gamification is highly effective for teaching about AMR across age groups. Aboalshamat et al. [58] found that a board game, *The Chancellor*, significantly outperformed lectures in improving and retaining AMR knowledge among students. Hale et al. [40] demonstrated that the e-bug game Body Busters effectively increased antibiotic knowledge and engagement in children aged 9–11 and was the most fun and engaging to play.

Implementation of technology into the educational process has proven to be very promising in several studies. Malli et al. [38] reported that the WHO online course improved knowledge and confidence in medical students. It was reported that pharmacy students rated antibiotic resistance as one of the most helpful topics in a blended learning course [37]. Portuguese WHO interventions with middle school students (14–16 years) also showed significant potential for improvement [59]. Ahmed et al. [42] demonstrated that their tech-supported AMR Roadshow for teens led to immediate and sustained knowledge gains, highlighting the long-term impact of interactive education.

The effectiveness of interactive methods such as peer education, gamification, blended learning, etc., can also be interpreted using Bloom’s taxonomy [56,57]. Traditional, lecture-based approaches often deal only with the lower levels of learning (remembering and understanding), while interactive approaches require students to apply, analyze and even create knowledge in new contexts. Peer-to-peer instruction, for example, forces students to restructure and explain concepts, fostering higher-order skills that improve retention. Similarly, game-based or scenario-based tools encourage application and analysis rather than memorization. This theoretical perspective helps explain why interactive strategies consistently show greater and more sustained gains in AMR literacy compared to didactic methods.

Taken together, interactive methods, peer education, blended learning, and gamified or creative tools offer promising strategies for improving AMR literacy in younger generations. However, evaluation metrics are often limited to self-reported knowledge rather than observed behaviors, making it unclear whether the improvements translate into responsible use of antibiotics in the real world. Future research needs to include longitudinal studies and behavioral measures to determine if the knowledge gains are lasting and if they meaningfully reduce inappropriate behaviors.

### 3.3. Publicly Available Tools and Programs to Improve Antibiotic and AMR Knowledge, Attitudes, and Behaviors in HICs

Efforts to combat AMR through education and policy have grown significantly worldwide. In 2025, the WHO released guidance on integrating AMR topics into school activities across subjects like science and health, using interactive methods to boost student engagement [60], and a WHO toolkit supports youth organizations in AMR awareness and advocacy [61]. HICs have implemented a number of youth-oriented initiatives to combat AMR through education and public engagement. In the European Union, the 2024 “Beat the Bug” campaign used the Fortnite creative platform to educate young people in several countries about antibiotics [62], while the e-Bug program offers interactive teaching resources in 23 languages and shows a significant increase in knowledge [63]. The Swedish STRAMA network supports the “Antibiotic Smart Sweden” initiative, certifies schools, and trains teachers [64]. The Spanish SWICEU project uses playful tools such as card games and escape rooms to inspire students at universities and secondary schools [65]. In the USA, the CDC’s “Be Antibiotics Aware” campaign provides materials for schools and youth organizations during Antibiotic Awareness Week [66], while Canada’s Pan-Canadian Action Plan includes school modules and youth ambassador programs [67]. Japan’s AMR Alliance coordinates national awareness efforts involving youth through civil society and educational activities [68]. Australia is integrating AMR issues into national science and health curricula and supporting national health authorities in providing resources for students and school participation in awareness campaigns [69]. These efforts reflect the growing recognition by international bodies such as the WHO and ECDC, national health authorities, and researchers that engaging younger generations in AMR education through innovative, culturally relevant, and evidence-based strategies is essential for sustainable antimicrobial stewardship.

Despite the documented knowledge gaps and behavioral problems, several studies in this review showed encouraging patterns of AMR literacy and demonstrated the effectiveness of targeted educational interventions. Cross-sectional analyses consistently found significant differences in knowledge acquisition between medical and non-medical student populations, with effect sizes favoring health professional education in all comparative studies included in this synthesis. Adherence metrics varied considerably by geographic and educational context, with some populations showing exemplary rates of adherence (e.g., 92% of German medical students) and a remarkably low prevalence of basic misconceptions (e.g., only 1% of UK students incorrectly believed that antibiotics treat viral infections). Most importantly, all six intervention studies showed statistically significant improvements in AMR knowledge compared to pre- and post-intervention assessments. In particular, interactive pedagogical approaches were pronounced in selected studies. Gamification interventions showed better knowledge retention than traditional didactic methods, while peer education models showed both immediate knowledge gains and sustained learning outcomes at follow-up periods of 6 to 12 weeks. These empirical findings provide an evidence-based framework for the development of targeted AMR education plans that go beyond traditional knowledge deficit models and incorporate behavioral science principles and interactive learning methods.

### 3.4. Limitations

This review has several important limitations that should be considered. First, most cross-sectional studies included in the analysis focused on undergraduate populations, leaving children and adolescents underrepresented despite their critical importance for early AMR prevention. While intervention studies addressed a wider age range, comprehensive understanding of AMR literacy across the full spectrum of younger populations will require future research involving school-aged children and adolescents. In addition, our analysis did not systematically examine whether misconception prevalence relates to cognitive developmental stages, which may explain some observed age-related variations in knowledge patterns.

Second, the diversity of study designs, survey instruments, and outcome measures limits direct comparability across studies and contexts. Most studies lacked validated, standardized AMR literacy assessment tools, and differences in instruments—for example, two studies adapting WHO questionnaires [31,36], while others used alternative surveys—restrict the ability to directly compare findings.

Third, many intervention studies were characterized by small sample sizes, short-term follow-up periods, and limited statistical power, making it difficult to evaluate sustained behavior change and generalize findings.

Fourth, cultural, educational, and systemic influences on AMR literacy were not consistently measured, leaving gaps in understanding how these factors interact with healthcare structures. Our review primarily focused on individual-level outcomes without systematically examining how broader cultural, regulatory, and healthcare system factors contribute to observed cross-country variations. Despite limiting the review on countries with well resourced healthcare systems, the variability in cultural, social, and regulatory factors might outweigh the influence of economic factors on AMR health literacy. Additionally, the exclusive focus on HICs introduces an inherent selection bias, limiting the understanding of global AMR literacy patterns and the transferability of findings.

Finally, the use of an AI-assisted screening approach (Claude 4.0) for the initial review of 4448 papers, while efficient, may have introduced systematic biases in study selection that differ from traditional human screening methods.

To address these limitations, future research should expand to younger populations, employ standardized and validated instruments, conduct longer-term follow-up with adequate sample sizes, evaluate culturally appropriate interventions, and systematically examine system-level determinants of AMR literacy patterns.

## 4. Materials and Methods

### 4.1. Literature Search

We searched the PubMed database for period between 2015 and June 2025. We used the following search terms: (health literacy) OR (attitude to health) OR (behaviour) OR (literacy) OR (knowledge)) AND (antibiotic) OR (Anti-Bacterial Agents) OR (antibiotic resistance) OR (resistance) OR (AMR) OR (bacterial resistance)) AND (child) OR (adolescent) OR (student) OR (young) OR (youth)) AND (pre-post intervention) OR (questionnaire) OR (pretest–post-test) OR (survey) OR (education)). This search yielded almost 15,000 papers, and after filter adjustment (in the last 10 years; Full text; English; Child: 6–12 years; Adolescent: 13–18 years; Adult: 19+ years; Young Adult: 19–24 years; Adult: 19–44 years), 10,052 papers remained for further selection. The terms (molecular methods) OR (covid) OR (laboratory experiments) OR (food) were further applied as exclusion criteria, and after the search run, 4448 papers remained.

### 4.2. Study Selection

In this review, we selected the existing research on AMR literacy among younger generations examining their knowledge, attitudes, and behaviors regarding antibiotic use and resistance in HICs. In addition, we examined various interventions proposed in the literature to improve health literacy regarding antibiotic resistance.

A literature search, AI analysis, and manual search, followed by a manual review of the papers, resulted in Figure 1. A list of the included studies/papers is provided in Table 1 and Table 2.

All 4448 paper citations were downloaded as plain text files and analyzed using an AI tool (Claude 4.0 Sonnet, Anthropic) on 11 June 2025. The following standardized prompt was applied: “From the 4448 selected articles from PubMed that you have in the file, select up to 100 most relevant articles that focus on antibiotic health literacy and antibiotic resistance in younger generations (children, students, adolescents). From the selected articles, choose those that contain research studies evaluating health literacy. Then select studies that include interventions or approaches (workshops, etc.) to improve health literacy among young people and studies that evaluate how these interventions have contributed to improving health literacy.” This process identified 95 papers meeting the specified criteria.

Subsequently, three researchers independently reviewed this selection by abstract and title and manually excluded 24 papers: 1 review, 1 duplicate, 1 in Spanish, 3 qualitative studies, 2 molecular biology studies, and 16 papers not focusing on AMR health literacy. The remaining 71 papers were screened for eligibility in full text. A total of 59 papers were removed for the following reasons: they were not from HICs, they were focused on parents or caregivers, or they contained a mixed population. Three researchers independently verified selection of the articles. Discrepancies were resolved through discussion. Fourteen studies from HICs were included in the review. To supplement the electronic search, we also conducted a search using related free text terms and included 5 studies/papers manually. In total, we included 19 papers/studies in the synthesis. We included both descriptive studies (surveys assessing literacy levels) and intervention studies (interventions aimed at improving knowledge and behaviors), as both are essential for informing effective AMR education strategies.

### 4.3. Data Extraction and Synthesis

We have collected information about (1) the source of the study: author’s last name and year of publication; (2) the setting and the demographic characteristics of participants; (3) the study design; (4) the main outcomes related to AMR knowledge, including knowledge, attitudes, and behaviors; and (5) the interventions proposed or implemented.

For cross-sectional studies (Table 1), we extracted data on study population size and characteristics, country of origin, main outcomes related to knowledge gaps and misconceptions about antibiotics, self-medication practices, adherence behaviors, and authors’ recommendations for educational interventions. For intervention studies (Table 2), we additionally collected information on the specific type of intervention delivered (gamification, blended learning, WHO online courses, peer education workshops, interactive sessions), study methodology (randomized controlled trial, quasi-experimental approaches, mixed methods), and outcomes in terms of knowledge improvement and behavior change. It should be noted that most of the cross-sectional studies summarized in Table 1 were conducted among undergraduate students, predominantly from medical and health-related disciplines. Consequently, Table 1 primarily captures the perspectives of young adults in higher education, whereas the intervention studies presented in Table 2 encompass a wider spectrum of younger populations across different educational levels, including adolescents and children.

For the 13 cross-sectional studies, data were synthesized thematically across all included studies, focusing on three main themes: (1) baseline misconceptions and knowledge gaps about antibiotic mechanisms and resistance, (2) self-medication and nonadherence behaviors, and (3) proposed educational interventions and strategic approaches.

For the 6 intervention studies, data were synthesized by comparing educational approaches and their effectiveness, focusing on two main categories: (1) traditional educational methods, including peer education workshops and blended learning approaches, and (2) virtual and technology-enhanced learning methods, including gamification, interactive digital tools, and online courses. The synthesis examined the outcomes of the interventions in terms of immediate knowledge gain, knowledge retention over time, and sustained learning effects in different age groups and educational settings.

This review was not prospectively registered in a systematic review registry. The review was initiated as part of a larger research project.

## 5. Conclusions

This review demonstrates that antimicrobial resistance (AMR) literacy among younger generations in high-income countries (HICs) remains suboptimal, despite the presence of well-resourced healthcare and educational systems. Across the included studies, persistent misconceptions—particularly the belief that antibiotic resistance develops within the human body rather than in bacteria and that antibiotics are effective against viral infections—were identified among both general student populations and students of health-related subjects. These knowledge gaps are closely linked to inappropriate practices such as self-medication, premature discontinuation of treatment, and the storage and sharing of leftover antibiotics. Studies revealing suboptimal knowledge of young generations in HICs call for the development of more effective educational and behavior change approaches. The variability in the results presented in selected studies points to the influence of cultural, educational, and systemic factors, highlighting the need for context-specific interventions.

Intervention studies show that targeted, age-appropriate educational strategies—including curriculum integration, gamification, peer education, digital learning, and creative outreach activities—are effective in improving knowledge and promoting more responsible antibiotic use. However, the evidence also suggests that biomedical knowledge alone is insufficient; behavioral patterns are strongly shaped by cultural norms, family influence, and systemic factors such as accessibility of antibiotics and regulatory environments.

Strengthening AMR literacy in young people therefore requires a multi-level approach. Education should not only focus on correcting misconceptions but also integrate behavior change principles, address informal social influences, and foster self-efficacy for antimicrobial stewardship. Early and sustained engagement of younger generations is essential, as they represent future patients, healthcare professionals, and decision-makers. By embedding AMR education into both formal curricula and wider community settings, HICs have the opportunity to cultivate long-term, responsible antibiotic practices that contribute meaningfully to global efforts against antimicrobial resistance.

## Figures and Tables

**Table 1:** Cross-sectional studies included in scientific synthesis.

Title (Author, Year)	Country	Population (N and Major Characteristics)	Main and Critical Findings Regarding AMR Literacy (Knowledge, Attitudes, and Behaviors)	Proposed Interventions
Antimicrobial knowledge and confidence amongst final year medical students in Australia (Weier et al., 2017) [26]	Australia	163 responses from final-year medical students from eight universities	General AMR awareness Students rated most factors as having a great impact on antimicrobial resistance (using the wrong antibiotic, prescribing when not needed, using an inappropriate dose, etc.). 94% were aware of antibiotic prescribing guidelines available in Australia. 56% of students reported that antibiotic guidelines were used in clinical practice at least 50% of the time. 97% reported that adhering to guidelines was important to reduce the risk of antimicrobial resistance. Australian medical students feel less confident in their knowledge of infectious diseases compared to other conditions.	Antimicrobial stewardship agenda should include the provision of additional training in antimicrobial prescribing to the future medical workforce.
Knowledge about antibiotics and antibiotic resistance among health-related students in a Saudi University (Akbar et al., 2021) [27]	Saudi Arabia	284 Clinical Laboratory Science, Nursing, and Pharmacy students	General AMR awareness 71.8% of the students knew the term antibiotic resistance. 63.7% were aware of the WHO agenda about antibiotic resistance. 82.7% reported resistance is a serious problem. Misconceptions 57.7% thought paracetamol is an antibiotic. 69.7% reported antibiotics work on viruses. 58.8% reported antibiotics were substitutes for anti-inflammatory drugs. Non-adherence 57.7% reported that it is appropriate to discontinue the antibiotic therapy once the symptoms are relieved. Self-medication 50.0% thought antibiotics could safely be used for any ailment without consulting the doctor.	Curricular contents must be reviewed and enhanced to suit the specific learning needs of students in terms of these concepts. Seminars and training should be provided to these students to further improve their awareness and knowledge of antibiotics.
Knowledge, attitude, behavior of the future healthcare professionals towards the self-medication practice with antibiotics (Benameur et al., 2019) [22]	Saudi Arabia	300 students (150 medical, 150 non-medical)	General AMR awareness Only half of medical students knew that antibiotics resistance is one of the most common side effects of antibiotic misuse. A smaller proportion of non-medical respondents were aware of AMR. Self-medication 40.8% of medical vs. 47.6% non-medical participants expressed a negative attitude towards keeping leftover antibiotics for future use. Medical students showed better knowledge but still engaged in risky behaviors. 79.9% of medical students knew self-medication was unsafe, yet 50.8% still practiced it.	Implementing tailored clinical training and enhancing medical students’ knowledge and raising their awareness is recommended. Authors highly recommend an early involvement of medical students in the antimicrobial stewardship program, not only as active learners but also as active medical team players.
A survey of awareness related to the use of antibiotics for dental issues among non-medical female university students in Riyadh, Saudi Arabia (Abu-Mostafa et al., 2017) [28]	Saudi Arabia	400 non-medical female university students	General AMR awareness 68% had poor scores on antibiotic efficacy, side effects, and resistance. Misconceptions 29.7% reported that antibiotics kill viruses. 68.8% reported that antibiotics relieve dental pain. 59.8% of the respondents agreed that the human body becomes resistant to antibiotics. Non-adherence 53% stop taking antibiotics when they feel well; 35.5% knew this bad attitude increases the risk of resistant bacterial strains. Self-medication 47.8% keeps antibiotics at home. 77.8% obtain antibiotics via prescription.	Community campaigns are recommended every university semester to educate students about the indications, efficacy, and side effects of antibiotics. Dentists, pharmacists, and other health professionals should participate in the education of patients.
Omani senior secondary school students’ knowledge of and attitudes to antibiotic resistance (Ambusaidi et al., 2022) [29]	Oman	952 senior secondary school students, ages 15–17, From 20 schools across Oman (urban and rural)	General AMR awareness Just over half of the students had come across the term antibiotic resistance. 17% expressed no knowledge of the concept of antibiotic resistance. Misconceptions 22% described resistance as developed by the human body rather than the pathogen. 62% believed that antibiotics were effective against viruses. Confusion between antibiotics and analgesics. Self-medication 22% would take antibiotics without prescription. 6% took antibiotics left over from a previous prescription. Non-adherence 38% knew to complete full antibiotic course; 48% would stop when feeling better.	Changes to the school curriculum are recommended to address misconceptions.
Knowledge, attitude and practice of antibiotic use among university students: a cross sectional study in UAE (Jairoun et al., 2019) [21]	UAE	600 non-medical students (NSs), 600 medical students (MSs)	General AMR awareness 76.2% have heard of resistance of bacteria, including 84.5% of MSs and 67.8% of NSs. Medical students scored better than non-medical students in attitudes, followed by knowledge and practice scores. Misconceptions 48% responded correctly to the question “Can antibiotics be used to cure infections caused by viruses?”, including 65.8% of MSs and 30.2% of NSs.	Adding courses on rational antibiotic use in the medical curriculum is recommended.
Antibiotic resistance: Italian awareness survey 2016 (Prigitano et al., 2016) [31]	Italy	797 persons, 666 young university students (median 20 years old)	General AMR awareness 89% of the respondents acknowledged that many infections are becoming increasingly resistant to treatment. 38% of respondents reported that a single person could do something to fight this problem. Misconceptions 22% of respondents correctly disagreed with the statement that “Antibiotic resistance occurs when your body becomes resistant to antibiotics, and they no longer work as well”. Self-medication 97% agreed with the use of antibiotics only when prescribed by a doctor.	Promotion of initiatives aimed at greater awareness about the use of antibiotics is recommended. It should be stressed that antibiotic resistance can be hindered when taking antibiotics in the appropriate way, completing the prescribed treatment to eliminate all bacteria, using these drugs only for bacterial infections, and avoiding misuse, which favors development of resistance.
Assessing the Knowledge, Attitudes and Behaviors of Human and Animal Health Students towards Antibiotic Use and Resistance: A Pilot Cross-Sectional Study in the UK (Dyar et al., 2018) [32]	UK/ England	255 students from 25 universities	General AMR awareness All students were aware that bacteria can become resistant to antibiotics. 95% felt that prescribing, dispensing, or administering inappropriate or unnecessary antibiotics is professionally unethical. Only 20% felt they had sufficient knowledge. Misconceptions Many assumed that humans (41%) or animals (44%) can become resistant. 95% perceived resistance to be a future problem. 25% of dentistry students thought that antibiotics were effective against colds. 1% thought that antibiotics kill viruses.	Continuing antibiotic education and other interventions is recommended.
Knowledge, attitudes, and practices related to antimicrobial resistance among undergraduate students at a large public university in 2020 (Tran et al., 2021) [30]	USA	605 individuals, undergraduate students	Misconceptions 32% of participants perceived AMR to only be a problem for people who take antibiotics often. 38% thought that antibiotics weaken the immune system. Non-adherence Many undergraduates have or would stop taking antibiotics before completing a full course because of side effects (44%) or feeling better (38%). Self-medication 23% would take and 13% would share antibiotics that had not been prescribed to the recipient.	Campus health services can play a pivotal role in educating all students about the safe use of antibiotics. Educating college students and others about the science behind AMR to equip them with self-efficacy to consistently practice antimicrobial stewardship behaviors is recommended.
Antibiotic literacy among Japanese medical students (Hagiya et al., 2020) [33]	Japan	661 undergraduate students at Okayama University Medical School	General AMR awareness 92.6% knew that antibiotics inhibit bacterial growth. Only 6.5% had heard about the AMR action plan. Misconceptions 46.4% of all students mistakenly believed that antibiotics could be used as an option for treatment of the common cold. 30.0% answered that antibiotics were effective in treating diseases caused by viruses. Approximately 20% of medical students expected physicians to prescribe antibiotics to them to treat the common cold. Non-adherence Around 60% of all students reported taking all the antibiotics they were prescribed.	Students should be provided with additional education in antimicrobial stewardship during the undergraduate period.
Appropriate antibiotic use and antimicrobial resistance: knowledge, attitudes and behaviour of medical students and their needs and preferences for learning (Wiese-Posselt et al., 2023) [34]	Germany	356 medical students	General AMR awareness 54% strongly agreed that the topic of AMR is relevant to students’ clinical practice. Only 5% of all participating students stated that they had acquired sufficient knowledge of antibiotic therapy and AMR thus far. Self-medication 10% of the sample had taken antibiotics without a doctor’s prescription. They had procured the antibiotic on their own, bought it abroad without a prescription, or used leftover drugs.	Improved student-centered teaching materials should be developed.
Antibiotic Use: A Cross-Sectional Survey Assessing the Knowledge, Attitudes and Practices amongst Students of a School of Medicine in Italy (Scaioli et al., 2015) [35]	Italy	1050 students of all the academic years of a School of Medicine, aged 20.98 (±2.94)	General AMR awareness 93.9% were aware that antibiotic resistance is a phenomenon in which a bacterium loses its sensitivity to an antibiotic. 98% knew that misuse of antibiotics can lead to antibiotic resistance. Misconceptions Around 20% of the sample stated that antibiotics are appropriate for viral infections. Non-adherence 94.8% of the students knew that it is mandatory to finish the full course of antibiotics even if the symptoms are improving. 15% of the students stop taking antibiotics when symptoms decrease. Self-medication 7.7% normally use leftover antibiotics without consulting a doctor. 16% claimed to buy antibiotics without medical prescription.	It would be advisable to introduce a specific course and training on antibiotics in the core curriculum of the School of Medicine.
Survey on antimicrobial resistance knowledge and perceptions in university students reveals concerning trends on antibiotic use and procurement (Leal et al., 2022) [36]	Canada	106 students from the Université de Montréal, without age, gender, or study program restrictions	General AMR awareness 82.1% participants said they had heard the term antibiotic resistance, 27.4% had heard of antimicrobial resistance, 76.4% of drug resistance, and 82.1% of antibiotic-resistant bacteria. Only 8.5% of participants said they had been informed about AMR through specific campaigns. Misconceptions 63.2% of participants answered that the statement “Antibiotic resistance occurs when your body becomes resistant to antibiotics, and they no longer work” was true. Non-adherence 4.5% of participants did not respect the correct daily dosage when taking a course of antibiotics; 12.3% stop using the medication once the fever has gone down. Self-medication 28.3% of students had used antibiotics without a prescription, 16.7% obtained antibiotics from the home medicine cabinet, 50% from the pharmacy, 26.7% from family, neighbors, or friends, and 6.7% from other sources.	There is a need to increase public awareness to better understand antimicrobial resistance’s theoretical basis.

## Data Availability

Paper selection/exclusion process for the current systematic review synthesis is available via Figshare https://figshare.com/s/23fd4183ddc4f5a6bc58 (accessed on 6 August 2025).
